# Supplementary material for: HCN2 channels in the ventral tegmental area regulate behavioral responses to chronic stress
Source: eLife. 2018 Jan 2;7:e32420. doi: 10.7554/eLife.32420 (PMC5749952; doi:10.7554/eLife.32420)
Supplement: Supplementary file 1. — Abbreviations: CMS, chronic mild unpredictable stress; EPM, elevated plus maze; FST, forced swimming test; NSF, novelty-suppressed feeding; OFT, open field test; SPT, sucrose preference test [file elife-32420-supp1.docx]

**Supplementary file 1**

| Measurement | Comparison | Statistics F value | Degrees of freedom | *p* value | Figure 1 |
| --- | --- | --- | --- | --- | --- |
| Body Weight | Control vs. CMS | 9.844 | 1, 25 | 0.005 | supplement 1A |
|  | C57 vs. DAT-tdTomato | 1.149 | 1, 25 | 0.295 |  |
|  | interaction | 0.0119 | 1, 25 | 0.914 |  |
| OFT-distance | Control vs. CMS | 0.248 | 1, 25 | 0.623 | supplement 1B |
|  | C57 vs. DAT-tdTomato | 0.0482 | 1, 25 | 0.828 |  |
|  | interaction | 0.970 | 1, 25 | 0.335 |  |
| OFT-center time | Control vs. CMS | 7.97 | 1, 25 | 0.010 | supplement 1B |
|  | C57 vs. DAT-tdTomato | 0.127 | 1, 25 | 0.725 |  |
|  | interaction | 0.0619 | 1, 25 | 0.806 |  |
| SPT | Control vs. CMS | 20.846 | 1, 25 | <0.001 | supplement 1C |
|  | C57 vs. DAT-tdTomato | 1.078 | 1, 25 | 0.310 |  |
|  | interaction | 0.166 | 1, 25 | 0.688 |  |
| EPM-open arm entries (%) | Control vs. CMS | 0.0144 | 1, 25 | 0.906 | supplement 1D |
|  | C57 vs. DAT-tdTomato | 0.233 | 1, 25 | 0.634 |  |
|  | interaction | 0.192 | 1, 25 | 0.665 |  |
| EPM-open arm time | Control vs. CMS | 4.567 | 1, 25 | 0.044 | supplement 1D |
|  | C57 vs. DAT-tdTomato | 0.209 | 1, 25 | 0.652 |  |
|  | interaction | 0.000354 | 1, 25 | 0.985 |  |
| NSF-novelty | Control vs. CMS | 8.110 | 1, 25 | 0.009 | supplement 1E |
|  | C57 vs. DAT-tdTomato | 0.187 | 1, 25 | 0.670 |  |
|  | interaction | 0.782 | 1, 25 | 0.376 |  |
| NSF-home | Control vs. CMS | 0.879 | 1, 25 | 0.359 | supplement 1E |
|  | C57 vs. DAT-tdTomato | 0.601 | 1, 25 | 0.446 |  |
|  | interaction | 0.0837 | 1, 25 | 0.775 |  |
| FST | Control vs. CMS | 28.072 | 1, 25 | <0.001 | supplement 1F |
|  | C57 vs. DAT-tdTomato | 0.186 | 1, 25 | 0.671 |  |
|  | interaction | 0.245 | 1, 25 | 0.626 |  |
